# Traffic sign classification using CNN and detection using faster-RCNN and YOLOV4

**DOI:** 10.1016/j.heliyon.2022.e11792

**Published:** 2022-11-26

**Authors:** Njayou Youssouf

**Affiliations:** Department of Computer Science and Engineering, Islamic University of Technology, Gazipur 1704, Bangladesh

**Keywords:** Convolutional neural network, Object detection, Faster R–CNN, YOLOv4, Traffic sign recognition, Traffic sign classification, GTSDB, GTSRB

## Abstract

Autonomous driving cars are becoming popular everywhere and the need for a robust traffic sign recognition system that ensures safety by recognizing traffic signs accurately and fast is increasing. In this paper, we build a CNN that can classify 43 different traffic signs from the German Traffic Sign Recognition benchmark dataset. The dataset is made up of 39,186 images for training and 12,630 for testing. Our CNN for classification is light and reached an accuracy of 99.20% with only 0.8 M parameters. It is tested also under severe conditions to prove its generalization ability. We also used Faster R–CNN and YOLOv4 networks to implement a recognition system for traffic signs. The German Traffic Sign Detection benchmark dataset was used. Faster R–CNN obtained a mean average precision (mAP) of 43.26% at 6 Frames Per Second (FPS), which is not suitable for real-time application. YOLOv4 achieved an mAP of 59.88% at 35 FPS, which is the preferred model for real-time traffic sign detection. These mAPs are obtained using Intersect Over Union of 50%. A comparative analysis is also presented between these models.

## 1 Introduction

Technology around Advanced Driving Assistance Systems (ADAS) is ever so increasing. To keep improving Intelligent Driving and Traffic Safety, Object detection plays an important role in the upcoming trend of self-governing cars [1]. Tesla inc. is the leading self-driving car manufacturer in the world and became the most valuable car company in the world during the writing of this paper. This shows the tremendous demand for self-driving cars in the auto industry. The ability of autonomous vehicles to detect, classify and act upon the different traffic signs can be a matter of human life. This means the technology ought to be accurate and fast to detect promptly. Because such a system has the benefits of saving lives and saving costs, developing and improving such a system is one of the main motivations for this paper. Using deep learning algorithms to design such a system is the main objective of our work. One of the advancements in scene understanding was OLIMP, introduced by Amira et al (2020) [2]. It made use of the multimodal dataset for a better perception of the environment. The different shapes and color coordination allow us to identify and differentiate between different signs. Though there are different aspects of traffic signs which make them differentiable, it is still a challenging task due to the problems of a variety of colors, shapes, environmental conditions, occlusion, illumination, etc. [3] Presents different classes of low-light image improvement algorithms and their improved versions to tackle the problem of illumination on images.

A system that aims at tackling these problems of recognizing traffic signs, should be able to detect traffic sing in an image or a video feed camera and then classify these images. In traffic sign detection, the algorithm has to scale since the vehicle of the device capturing might be at a different distance at different times. We explored the different filter sizes to identify the effect of size on the performance of the models. In this paper, we look at the different classifications that exist and we proposed our CNN that is lighter and faster but slightly less accurate. We also explored the algorism for real-time object detection.

Our contributions can be listed below:

1. We introduced a lighter and faster CNN with a highly acceptable accuracy of 99.20% for traffic sign classification from GTSRB.
2. We Fine-tuned the Faster R–CNN and YOLOv4 network architecture to train and detect traffic signs from GTSDB.
3. The original GTSDB dataset divided the data into only 3 categories i.e., prohibitory, mandatory, and danger. Our paper used this dataset not only to detect the 3 categories but to detect all 43 categories of the German traffic sign individually.
4. A comparative analysis between these two models to understand each model's performance and compromise over the other.

In the next section, 2, we do a literature review and understand what existed, what exists, and why they need improvements. Then in section 3, we discuss the methodologies that were used in this paper and all implementations. Experimental Results, IV, is the section where we compared our obtained results to the state-of-the-art algorithms. And also, a comparative analysis between our used models. And we concluded our work in section 5.

## 2 Related works

First, the task of Traffic Sign Recognition (TSR) can be subdivided into two main categories: Traffic sign detection (TSD) and Traffic sign classification (TSC). Many CNN networks have been designed in recent years to perform object classifications and detections. Comparing the different results achieved by different researchers is a challenging task because of the use of different datasets, GTSDB, BTSD, etc. which have different properties like the number of training datasets, and quality of training images, which affects considerably the performance of the network (Fang et al., 2018) [4]. proposes a novel technique for classifying land use based on images captured by different users under different conditions. Nevertheless, over the years new techniques have been developed to improve the performance of classification and real-time object detection by reducing network size, increasing accuracy, and reducing detection time.

Before the advent of CNN, object classification and recognition were a bit more difficult and expensive to achieve. Maximally Stable Extremal Regions (MSER) were used in combination with Class-Specific Extremal Regions (CSER) in [5] to detect text-based regions and recognize matching text fields, with CSER and MSER attaining a precision of 80% and 50% respectively. Traditional network for image classification has been developed over the years, AlexNet [6] introduced in 2012 was revolutionary in image classification. It improved the traditional CNN. It made use of the importance of the ReLU activation function over the tanh function. In ImageNet Large-Scale Visual Recognition Challenge (ILSVRC), a contest for best performing networks, AlexNet [6] had the best performance [6]. was used as the standard model for image classification. VGG [7] (2012), which is considered to be the next step after [6], focused on a particular aspect of CNN, which is the depth of the network. Also decreased the size of the network and increase accuracy. ResNet [8] also decreased the amount of computation compared to VGG [7] by a factor of 5. Other algorithms [9] did not employ. CNNs are good for classification at a high level because of their invariance properties [10].

Application-specific classifications of objects like traffic signs [11, 12], and [1], have been introduced with different techniques to achieve better accuracy. L. Chen et al (2017) [11] presented a Combined Convolution Neural Network. It consisted of two separate CNN, one for superclass recognition which consisted of 6 classes, and the other CNN for subclass recognition which consisted of 43 classes. Then combined the result of both CNN to determine the final label using vector summation. The framework in [11] achieved a recognition accuracy of 95.6%. However, the proposed framework time cost is 2.7 ms. Though the result was acceptable, the classification time was too slow for real-time application. Kedkarn et al. 2015 [1] also use techniques like SVM to classify traffic signs from GTSRB. In object detection, the methodology developed so far can be classified into mainly two categories: one-stage detection and two-stage detection approaches. R–CNN [13] used Region proposals to extract the features and also improved precision [13]. uses selective search to generate all the proposed regions, about 2000 regions. This selective search slowed down the network. A better network has been designed soon which achieved better performance. SSP Net allowed the detection of input images of different resolutions. Fast R–CNN [14] used a parameter aggregation network (SPP) to improve the precision compared to R–CNN. The Faster R–CNN [15] discarded the selective search method and used the RPN for feature extraction, which saw considerable improvement in the performance of the network. Abdullah et al [16] proposed a system to detect vehicles by using deep learning algorithms [16]. used mainly R–CNN and fast R–CNN which achieved an mAP of 64% and 75% respectively, the high mAP in [16] is because the detection and recognition are that of vehicles rather than traffic signs which are much smaller and more similar [15]. also uses ROI pooling to scale its input. The network looks at the image twice before it completes. In one-stage detection, the image is looked at by the network only one which makes it faster and more suitable for real-time application. Liu et al. [17] introduced SSD. SSD [17] uses a grid system to divide the image into multiple sections. In each independent grid, that grid is responsible for detecting objects in its region [17]. has better accuracy compared to YOLO [18] which combined detection and classification into one CNN. Which makes [18] faster but less accurate.

Then YOLOv2 [19] and YOLOv3 [20] were an improvement on the previous versions [20]. use the DarkNet53 which is a bigger network, 53 CNN, than [19] DarkNet19 of 19 CNN, therefore increasing the accuracy of [20]. YOLOv4 [21] (2020) is the latest in the YOLO series at the time of writing paper. YOLOv4 [21] used CPSDarkNet53 and which makes it capable of detecting smaller objects. Can be used in1080 Ti or 2080 Ti GPU for training. A comparison of these networks on the COCO 2017 dataset is given in Table 1.

**Table 1:** Comparison of different networks for object detection.

Model name (backbone)	Speed (ms)	COCO mAP (%)
YOLOv4 CSPDarknet-53	38	43.5
SSD VGG-16	43	39.5
Faster R–CNN ResNet50	92	34.9
YOLOv2 DarkNet-19	25	21.6
YOLOv3 DarkNet53	45.5	42.4

## 3 Methodology

### 3.1 Dataset collection and organization

For the classification module, the German Traffic Sign Recognition Benchmark, GTSRB, was used. This dataset consists of about 51822 images stored in the Portable Pixmap (ppm) format. About 39209 images were used for training and 12630 were used for validation. The sizes of these images range from 15 × 15 to 250 × 250 pixels. The Region of Interest ROI, for each image, is provided and was used. The annotation of these images was also collected in comma-separated values (CSV) files. There was a total of 43 different classes in the dataset. Samples of some classes in our dataset are shown in Figure 2.

**Figure 1:**
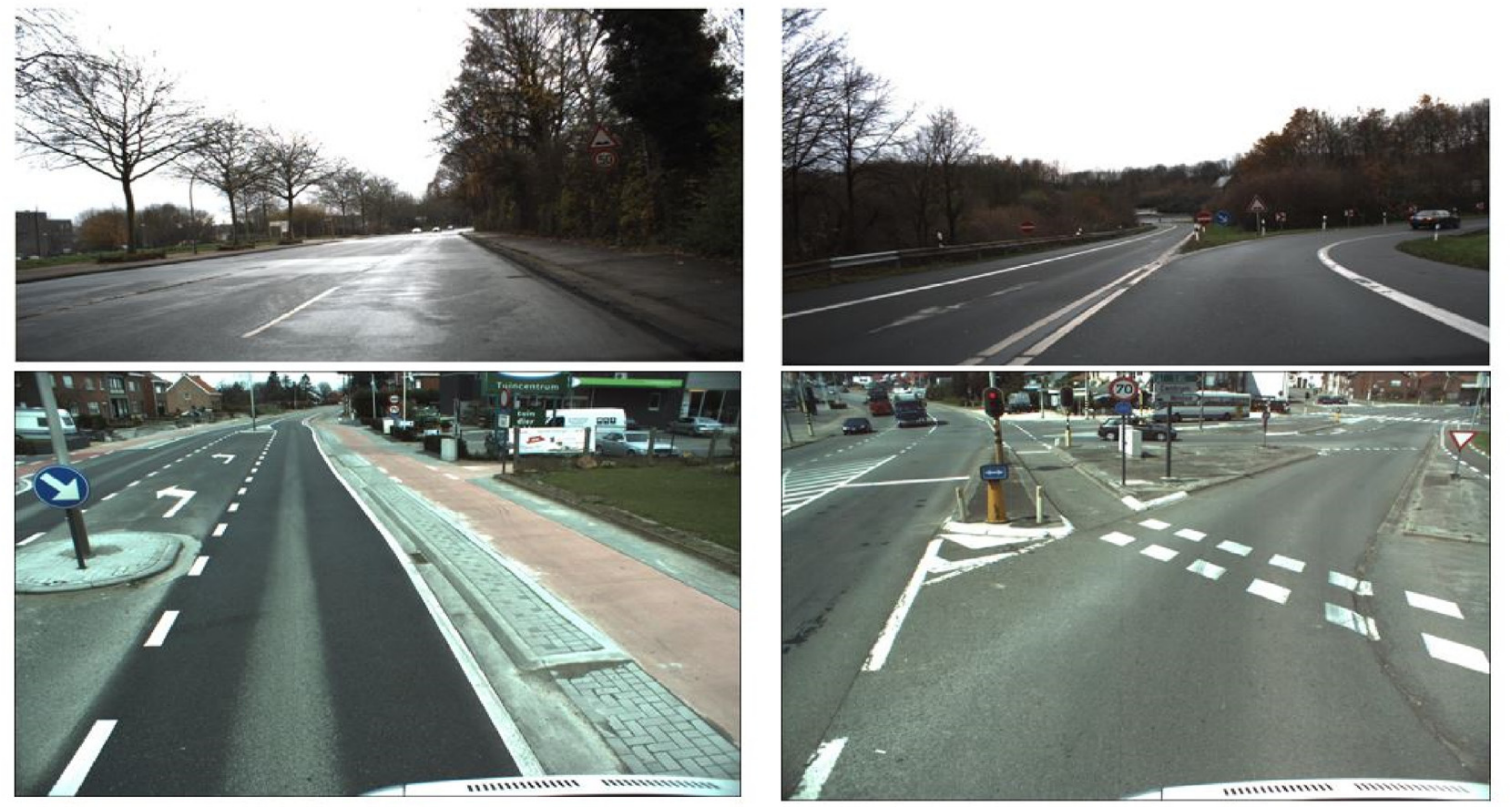
a) Top row shows images from GTSDB dataset and b) Bottom row shows images from Belgium traffic sign dataset.

The dataset that was used in the detection module of this work is the German Traffic Sign Detection Benchmark dataset (GTSDB), it consists of 900 images all of size 1360 × 800 pixels, stored in ppm format. The labels of these images were also collected in a CSV file which contained the file name and the information about the ground truth location of the actual traffic sign on the image. This dataset was divided into two, the training and the validation sets. The training set consisted of randomly selecting 600 images among all the images and the remaining 300 images were used for the validation set. Since the training set was not large enough, only 600 images, additional 300 images from the Belgium Traffic Sign Dataset (BTSD) which contained images of traffic signs were also collected, making a total of 900 images for the training set and 300 images for the testing set. The image size is 1628 × 1236 pixels for every image from BTSD and 1360 × 800 for images from GTSDB. Then these images’ ROIs were manually labeled in the Pascal VOC (Visual Object Classes) format. Any of these images may contain more than one traffic sign in the same image. The images were taken under different lighting conditions, different angles of the sign, and occlusion in some cases, see Figure 1. The GTSRB, GTSDB, and BTSD datasets used in this paper are publicly available for use.

**Figure 2:**
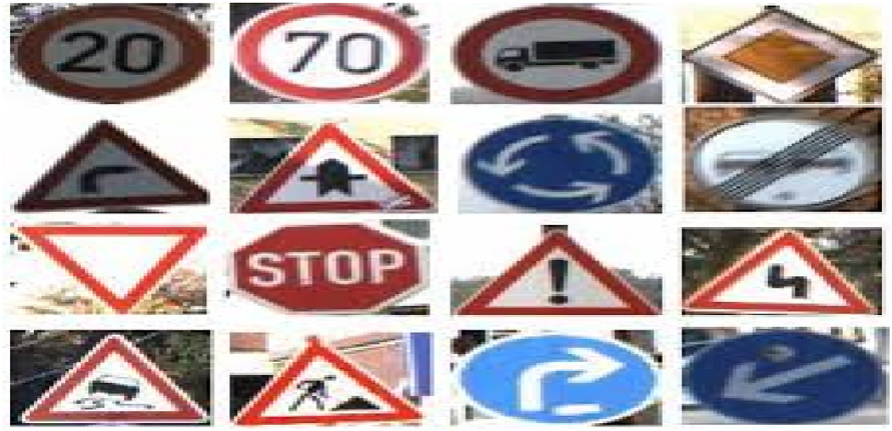
Samples of images from GTSRB for classification.

### 3.2 Data pre-processing

**Figure 3:**
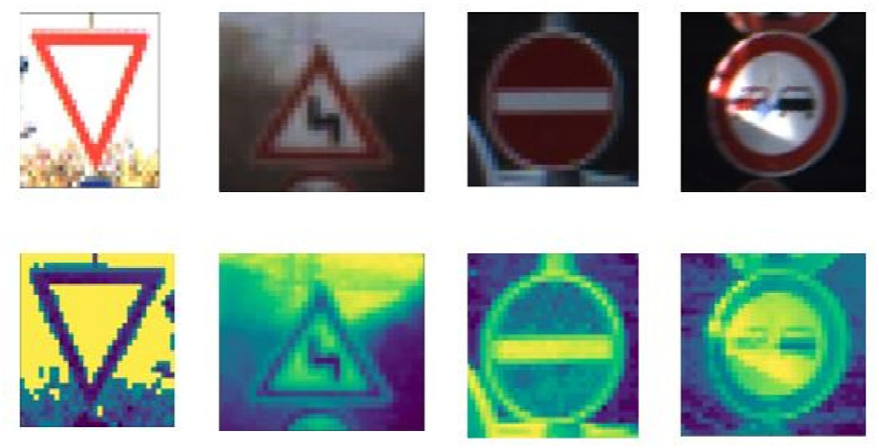
Preprocessing images samples.

In the detection phase, the image ROI annotations were converted to Pascal VOC format from the CSV format annotations and the same was also done for the classification model. In the classification phase, the images were resized to 32 × 32, then converted to grayscale, and then the images were normalized. Resizing of the images was because the image size of the dataset ranged from 15 × 15 to 250 × 250 pixels. In addition, since our CNN receives only input images with the same size, all images must be resized to a specific size before being passed into the network. Then converting the images to grayscale was because the colors of the images are not a very important determinant factor for the classification of the image. And it also reduces the complexity of processing the CNN. There is significant scientific value in enhancing system robustness in adverse weather conditions and ameliorating image quality [22]. Then the normalization of the images was done by dividing each pixel by the maximum pixel. This ensures that the input pixels have analogous data distribution. It also makes convergence faster while training the network. The results of this preprocessing are shown in Figure 3. The distribution of the images across the 43 classes is shown in Figure 5. Image augmentation techniques like shifts, Brightness, and zoom were used to even the distribution of the number of images per class to improve the classification accuracy and reduce bias. The two-stage detection network is more accurate in the detection of bounding boxes and class objects.

### 3.3 Traffic sign classification module

For the classification module, the GTSRB was used. The dataset was split in a ratio of 20% for testing, 20% for validation, and 60% for training. The number of images in each class is not evenly distributed therefore an augmentation technique was used to increase the number of training sets.

**Figure 4:**
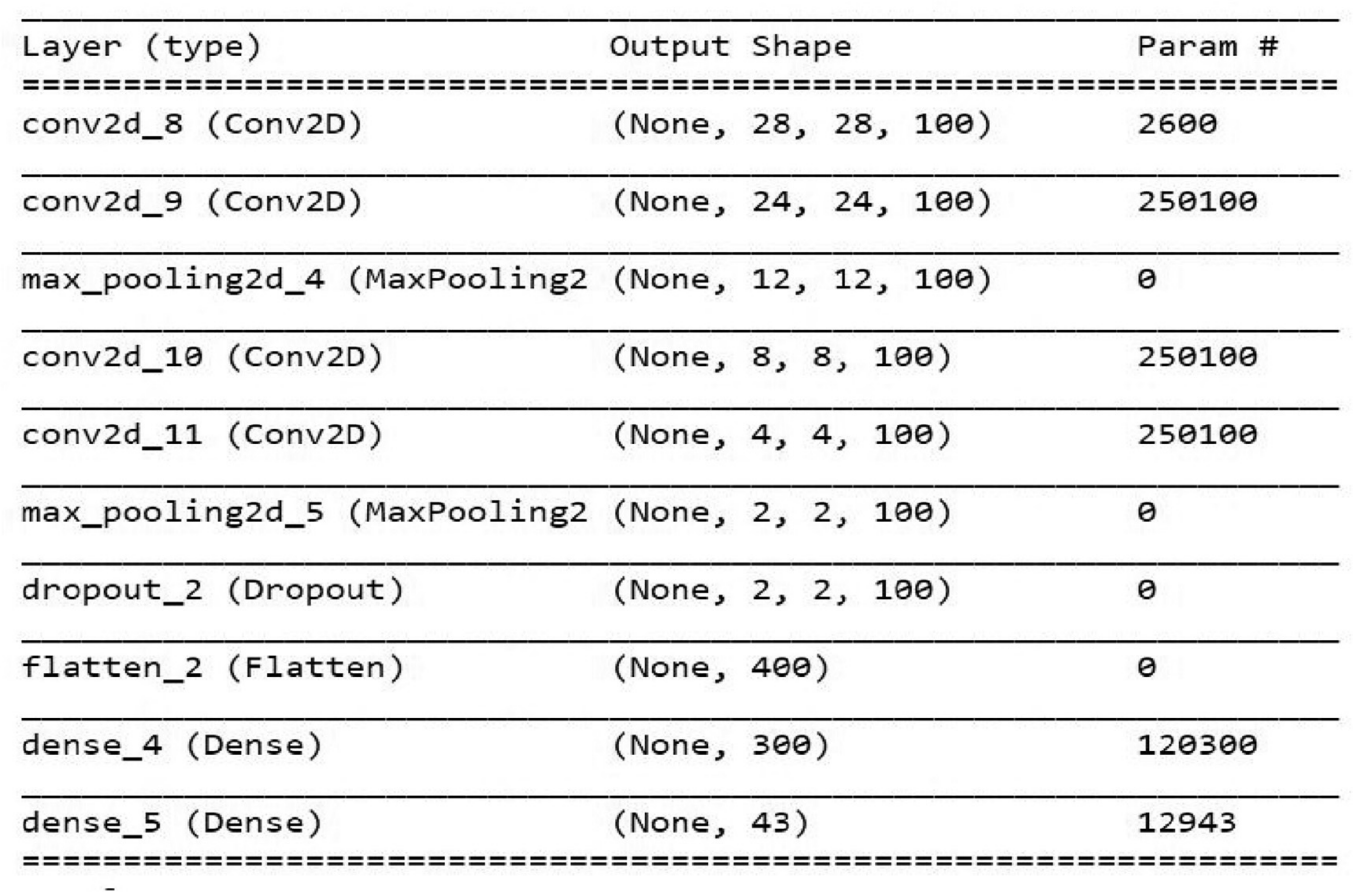
CNN architecture for traffic sign classification.

**Figure 5:**
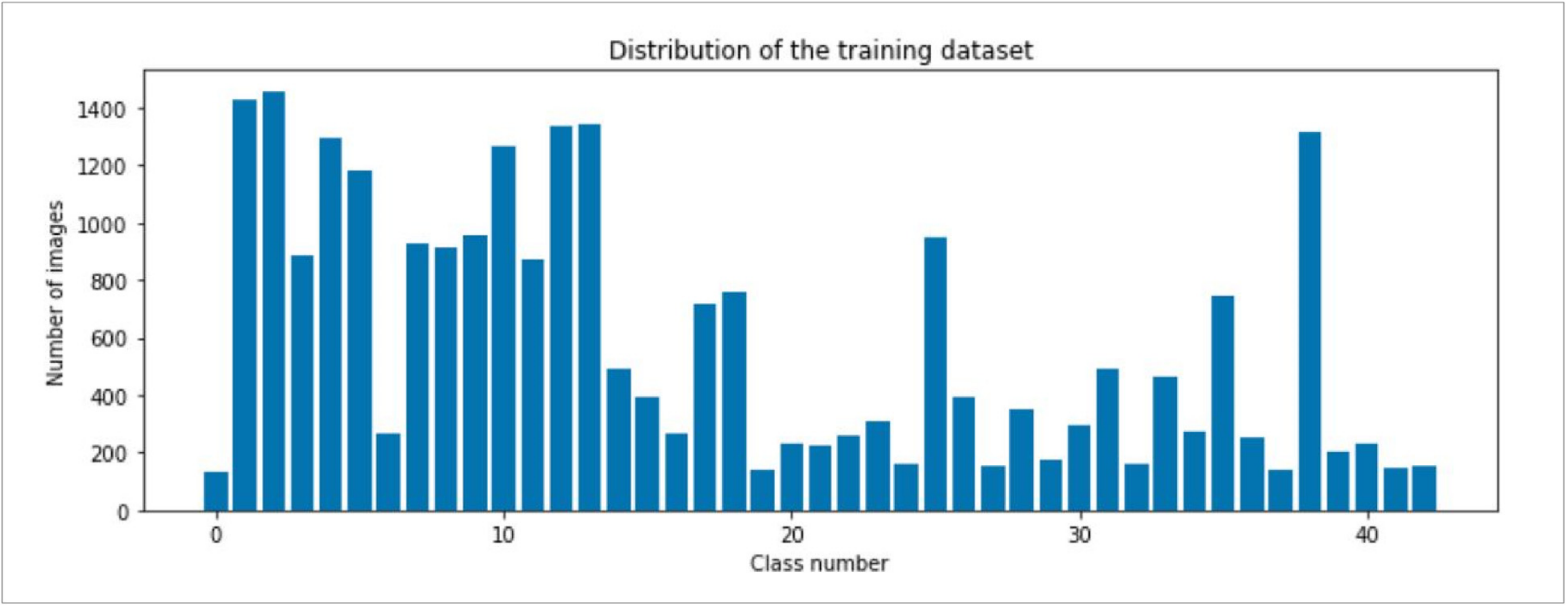
Distribution of images across the 43 different classes.

**Figure 6:**
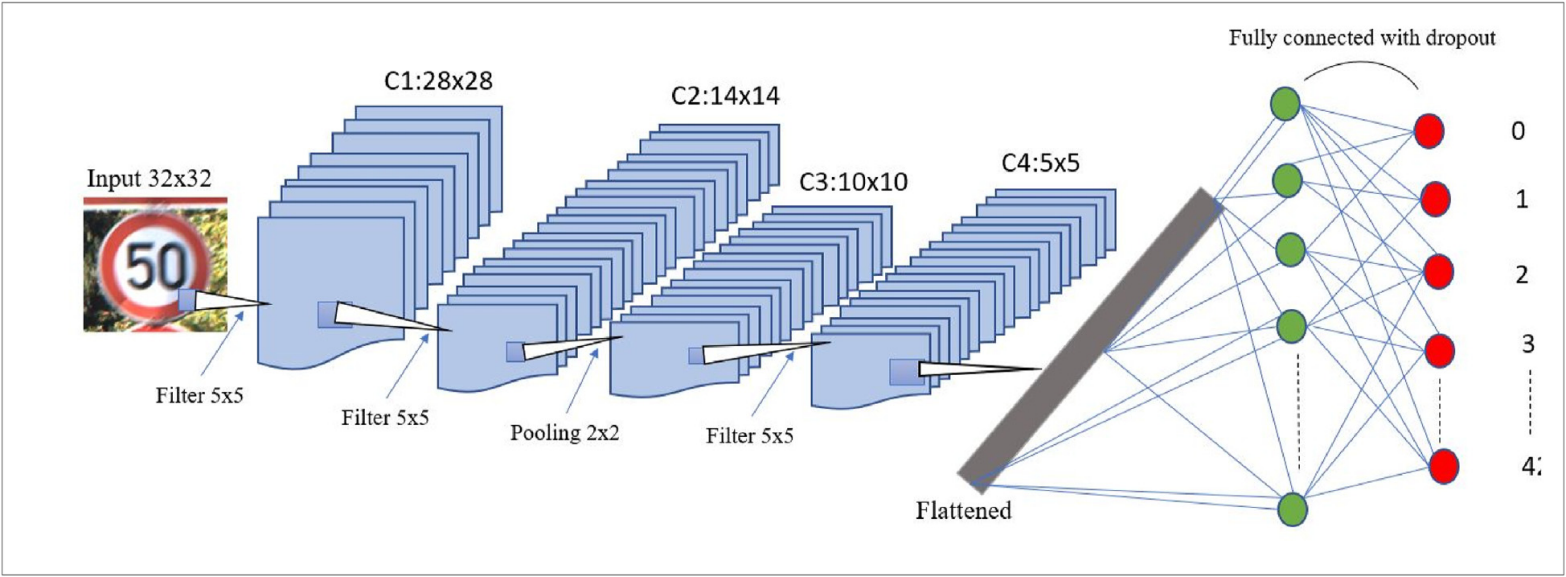
Convolutional neural network used for classification.

The class ‘speed limit 20 km/h’, represented by 0 in Figure 5, has 210 images while the class ‘Speed limit 50 km/h’, represented by 2, has 2250 images. Because of these discrepancies, the model may become biased towards the class with more images. The different augmentation parameters included random rotating, stretch, and flips. These augmentation parameters are used to balance the dataset to reduce bias. From the dataset, the images occur successively for the same class and similar images occur one after the other, because of this presence, random shuffling was performed on the dataset to avoid fluctuation of the training and loss functions. A Convolutional Neural Network CNN was constructed to do feature extraction and classification on the training set. The convolutional filter size was 3 × 3 since it was better for smaller objects. A ReLU activation function was used in different hidden layers and the categorical cross-entropy loss function together with the Adam optimizer and a learning rate of 0.001 was also applied. A summary of the architecture of the CNN for classification in Figure 4, shows the different hidden layers and filters and pooling applied to the neural networks. The network architecture that was developed is shown in Figure 6. The proposed CNN for traffic sign classification was developed using the python framework, TensorFlow, in addition to other libraries.

### 3.4 Traffic sign detection module

Most of the prominent state-of-the-art object detection techniques are divided into two: One-stage detection and two-stage detection. Examples of two-stage detection techniques are Faster R–CNN (Region-based CNN), and Mask R–CNN, which uses RPN. Examples of one-stage detection include You Only Look Once (YOLO) [18], EfficientNet [9], and SSD (Single Shot MultiBox Detector) MobileNet. [18], compared to an SSD which has a high inference speed. So, our objective is to reduce the compromises between speed and accuracy that exist between the one-stage and two-stage object detection techniques. The two techniques followed in this work are YOLOV4 [21] and Faster R–CNN [15] object detection techniques.

### 3.5 Faster R–CNN

Faster R–CNN [23] is a deep convolutional network used for object detection [23]. proposed a model that is made up of two modules: the fully convolutional neural network called Region Proposal Network (RPN), which proposes region boxes, and the next module is the detector, which classifies the object. In the first module, there is a fully connected convolutional neural network that is used for feature extraction. This CNN will extract the main feature of an image and the output of this CNN is a feature map. The feature map is then passed to another network layer, the Region Proposal Layer, which is responsible for proposing the potential regions where an object might exist and giving the class and the probability or score of that particular proposed region belonging to that class. For every other location on the feature map, a sliding window is used in the RPN (Region Proposal Network). Different bounding boxes are used for each location. 3 scales (128,256, 512) and 3 aspect ratios (1:1, 1:2, 2:1) are used in each location for the proposed regions. This increases the generalization of the network. It checks which of these locations contain objects and then these objects will be passed to the next network for detection. Non-max Suppression is used to remove overlapping regions. For detection, it goes through an ROI pooling layer, then each ROI feature vector goes through a CNN, and then through SoftMax where the final prediction will be made. This network architecture was fine-tuned to detect traffic signs from GTSDB. Fine-tuning is the process of using pre-trained weights of a model for recognition on a new dataset. The first few layers of Faster R–CNN can extract generic features (Faster R–CNN [23], 2017) since it is trained with a big dataset. We used Faster R–CNN architecture trained on the PASCAL VOC 2007 dataset (Shaoqing et al. (2017)) as the pre-trained model. As our dataset was relatively large (39,186 images) compared to the PASCAL VOC 2007 dataset, we theorize that fine-tuning the first layers of the Faster R–CNN rather than the later layers would improve performance. We fine-tuned the earlier CONV + ReLU layers of the Faster R–CNN by decreasing the depth to 32 and the filter size to 3 × 3, because our training images are smaller (32 × 32 pixels), thereby decreasing the network size and increasing performance. We also initialized the FC + ReLU layer to enable training from scratch for multi-class classifications. Random Adjust Hue, Random Adjust saturation, and random Adjust Contrast were used to minimize the effect of an unbalanced dataset in training.

### 3.6 YOLOv4

**Figure 7:**
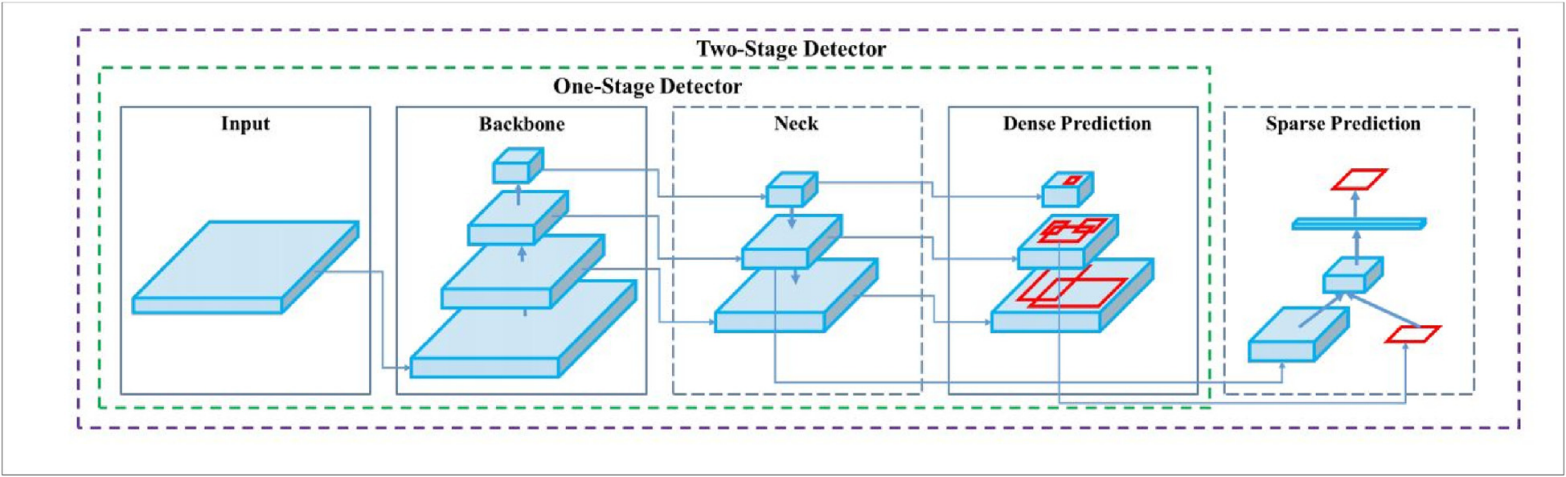
Object detector architecture.

The Single-Stage Detection used is the YOLOv4. A faster speed and better accuracy can be achieved by YOLOv4 which uses several network architectures [21], Figure 7. The Input section in the figure represents the input of the image which can be of variable resolution from 32 × 32 to 512 × 512 or more. Some data augmentation process occurs to generalize the network so it can recognize objects of different sizes. Then it is passed to the backbone section, which is where the feature extraction occurs. This backbone can be the different networks of VGG, EfficientNet, darkNet53, or ResNet. In Figure 7, the backbone used is CSPDarknet53, which separates the layers into different parts, of which one to passes through convolution and the other part does not, and then the results are combined, which improves the learning capabilities of CNN. The next section of the YOLOv4 architecture is the Neck. The Neck adds more layers from the backbone to the dense prediction block. It serves as an aggregation layer. In YOLOv3, the Feature Pyramid Network is used to extract the features of different resolutions from the backbone, but in YOLOv4, the Path Aggregation Network (PANet) is used for this purpose, which gives higher accuracy. Then the Spatial Pyramid Pooling (SPP) used in R–CNN is also used to map any size input to a particular fixed output size. The final section is the head section (Dense Prediction), similar to YOLOv3, which is responsible for locating the bounding box coordinates (x, y, w, h) from the previous layers and classifying the image section within the bounding box. It concurrently predicts numerous bounding boxes and the likelihood of those bounding boxes to belong to a specific class. The network uses Intersection over Union (IoU) in Eq. (1) to detect the boundary boxes for the region proposals, where A_gt_ is the background truth and A_p_ is the predicted.

(1) $$IoU=\frac{A_{gt}\cap A_{p}}{A_{gt}\cup A_{p}}$$

YOLOv4 was fine-tuned to detect traffic signs from GTSDB. YOLOv4 has been pre-trained on PASCAL VOC 2007 with acceptable mAP (mean average precision). The purpose of fine-tuning is to decrease the time of training and increase accuracy and also reduce the cost of training. It increases the data space. The fine-tuned network was trained on the GTSDB dataset. We fine-tuned the YOLOv4 architecture which was the pre-trained model. The fine-tuning technique was applied to the state-of-the-art object detection algorithm YOLOv4, trained on the COCO dataset, to specifically suit the needs of Traffic Sign recognition and detection using some custom dataset. The weights of the pre-trained YOLOv4 model, convolutional layers, were kept the same as the pre-trained model. The input resolution size was increased to 614 × 614 making the YOLOv4 model easier to detect smaller objects. The weights of the dense layers were updated by passing new data. The pre-trained YOLOv4 was trained on the COCO dataset, which was a dataset of 80 different classes for detection. In our model, there are only 43 classes and it uses the same weights in the convolution layers which makes the model much faster and also increases its performance of the model.

## 4 Experimental results

**Figure 8:**
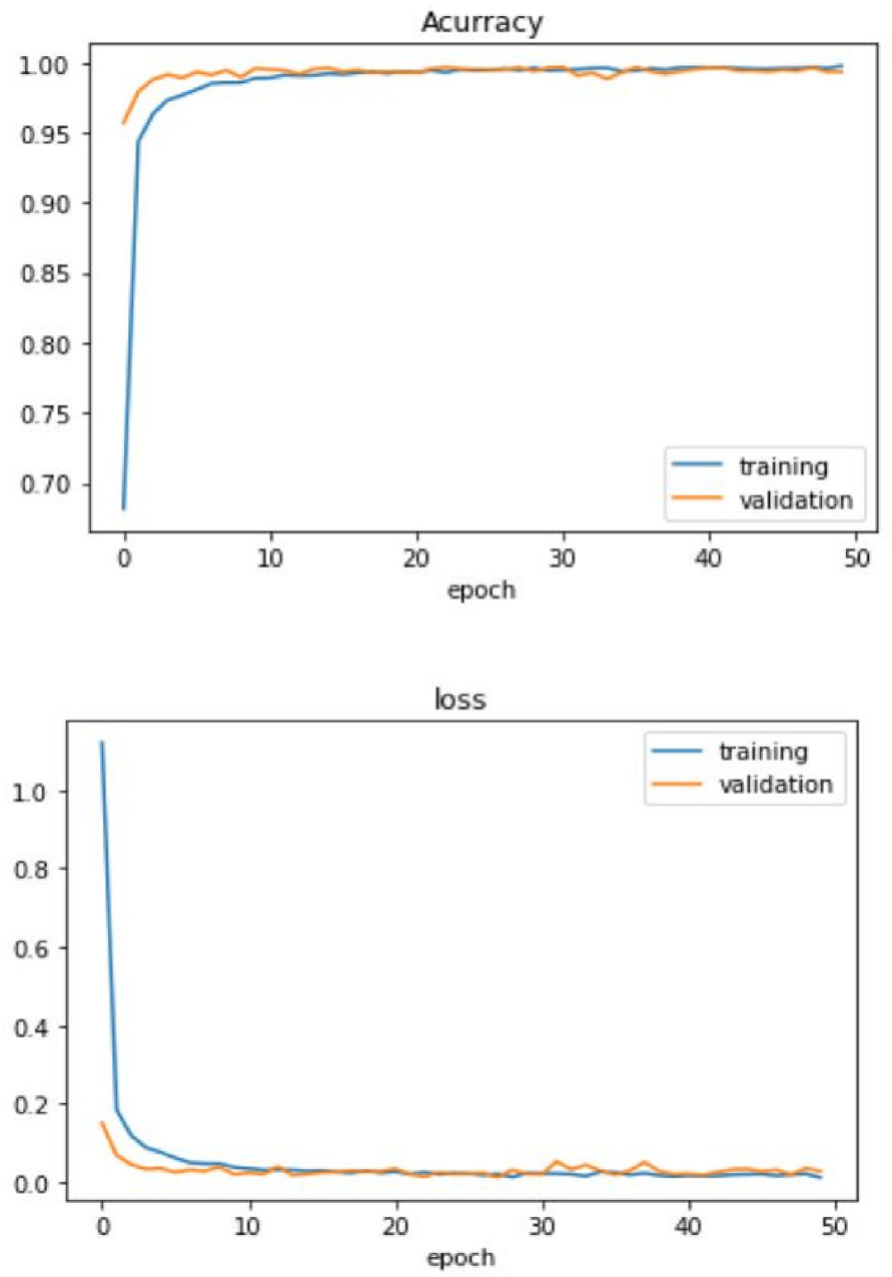
Accuracy and loss function for our CNN classifier.

For classification purposes, the developed CNN shown in Figure 4 achieved a good accuracy of 99.20% on the GTSRB test dataset. The accuracy and loss graphs for training and validation of the model are shown in Figure 8, Our model took approximately 6.63 s to classify all the images in the test set. The test set consists of 12360 images; therefore, the model takes 0.14 ms to classify a single image. It is considered fast for applications that are not real-time. The comparison between our CNN and other state-of-the-art CNN image classification is given in Table 2 [12, 16, 24]. The accuracy of our CNN is less than some of the prominent CNN for classifications but considering that the number of parameters for our network is 1/5 that of MCDNN [24] and 1/38 that of Co. CNNs [16]. To prove the robustness of our network, some of the successful classifications under different tough conditions, low and high illumination, occlusion, and blurring, are provided in Table 4.

**Figure 9:**
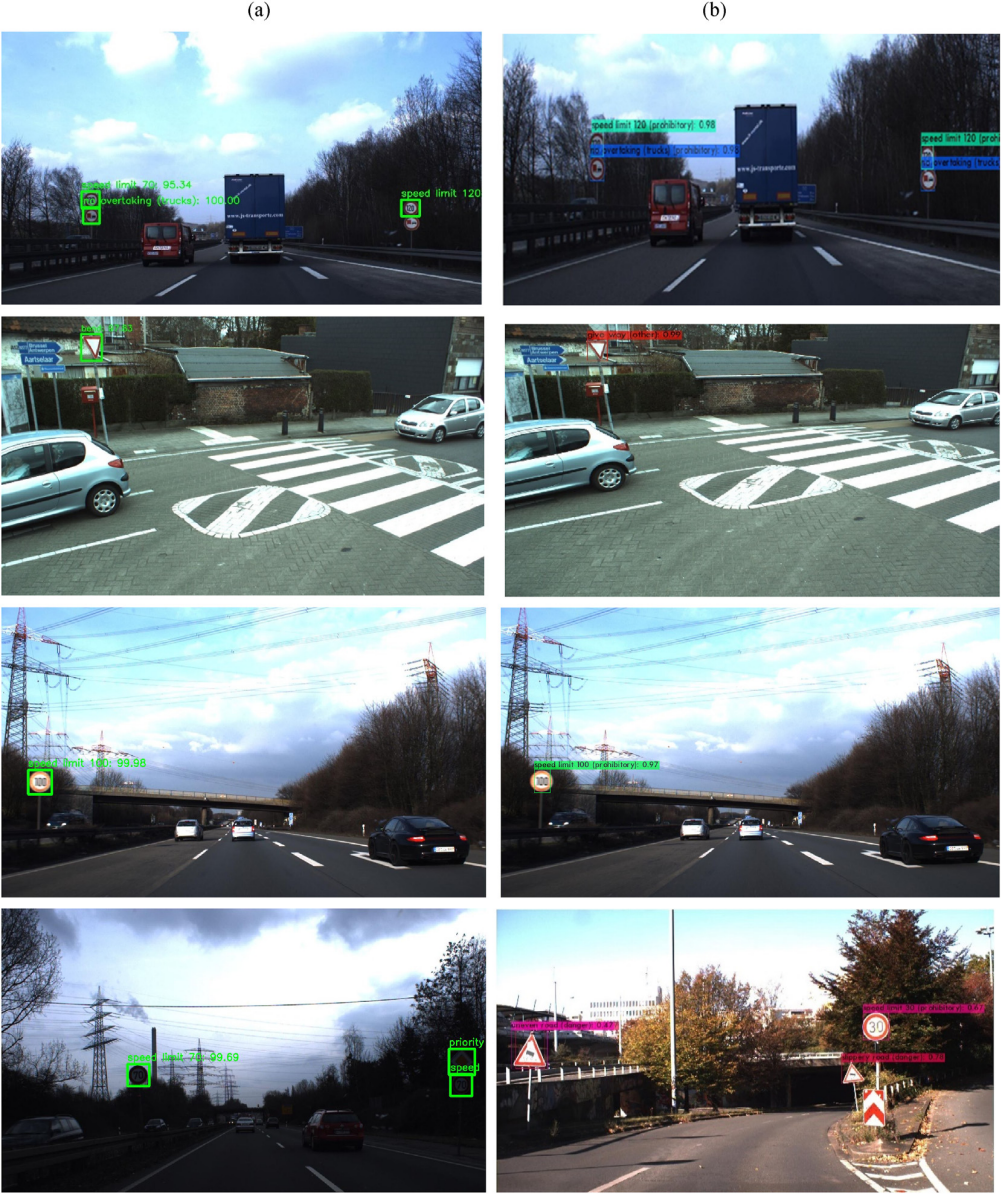
A comparison of the results from Faster R–CNN and YOLOv4. The first column (a) is from Faster R–CNN and the second column (b) is from YOLOv4. From the first row, Faster R–CNN misses the *No overtaking sign* and falsely classifies *Speed limit 120* as *speed limit 70*. YOLOv4 correctly detects and classifies all the signs. From the second row, Faster R–CNN miss classifies *give way sign* as *bend sign*. YOLOv4 correcly classifies the *give way* sign. From the third row, both Faster R–CNN and YOLOv4 correctly classify the *speed limit 100 traffic sign*. The last row shows how Faster R–CNN result in low light and YOLOv4 results in bright light. Both the models perform well in conditions of low light and bright light.

**Table 2:** Comparison between our CNN and other states of the art CNN classifier on GTSRB.

Model name	Time	Loss	Accuracy	No. of Parameters
Our CNN	6.631 s	0.031	99.20%	0.8 M
Enet-V1 [12]	7.794 s	0.064	98.69%	0.9 M
Enet-V2 [12]	3.090 s	0.2642	96.78%	0.31 M
MCDNN [24]	11.4	0.024	99.46%	38.5 M
Co. CNNs [16]	-	-	99.35%	5.22 M

To evaluate the performance of the different methods used in this work for object detection, Faster R–CNN, and YOLOv4, an experiment was conducted as mentioned above and then the resulting weights from these object detection techniques were evaluated on a real-time video feed from the street of berlin. The process of labeling, training, and evaluating these models was done on a PC with a 3.4GHx Intel CPU, 16G RAM, and 4G NVIDIA GeForce 1060 GPU. CUDA was used to improve the performance and speed of the model. The fine-tuned Faster R–CNN reached an mAP (mean Average Precision) of 43.26% on the GTSDB, with a speed of 6 FPS (Frame per Second) and the YOLOv4 reached a much higher mAP of 59.88% on the same dataset. Table 3 shows the comparison between these models. The Faster R–CNN is too slow and would need improvements to be used in real-time applications. A video, Video 1, was fed to the inference of both model and the traffic that was in the video, along with whether the model correctly detected and recognized it is given in Table 5.

**Table 3:** Comparison between our developed models.

Model	mAP	Speed (FPS)
Faster R–CNN	43.26%	6
YOLOv4	59.88%	35

**Table 4:** Results of our CNN under certain conditions of ambiguity, very low illumination, low illumination, blurriness, occlusion, and high illumination. All were correctly predicted.

Input	Predicted	Confidence
	Speed limit (30 km/h)	100.0 %
	Speed limit (100 km/h)	98.67%
	Speed limit (80 km/h)	67.18%
	Slippery road	99.77%
	Slippery road	98.28%
	Speed limit (30 km/h)	99.96%

**Table 5:**
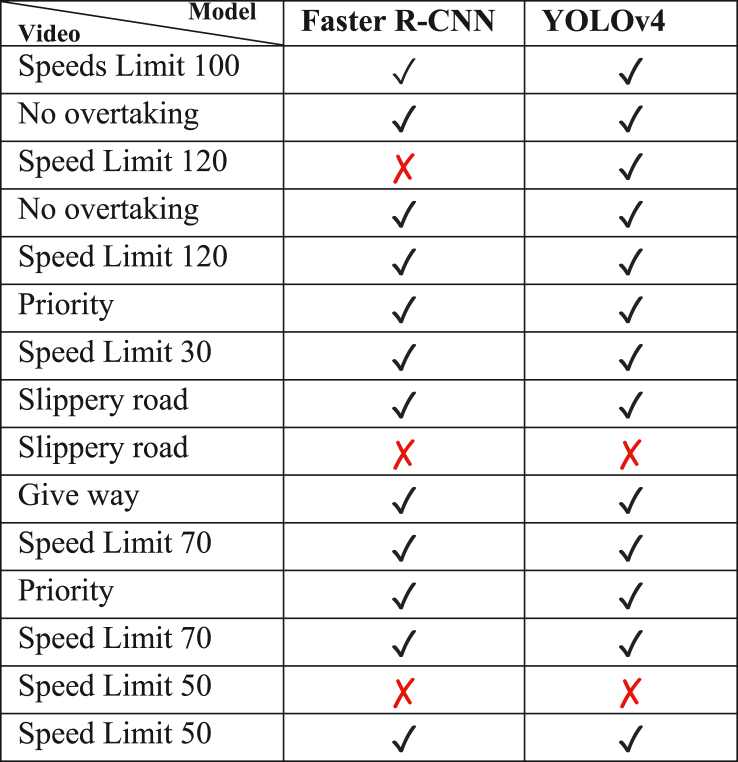
Performance of the models on Video 1.

Supplementary video related to this article can be found at https://doi.org/10.1016/j.heliyon.2022.e11792

The following is the supplementary data related to this article:

Multimedia component 1 Multimedia component 1

## 5 Conclusion

In this paper, a novel CNN architecture was designed for traffic signed recognition and was tested on the GTSRB dataset and achieved a very high accuracy of 99.20% with minimal loss, which is comparable to the state-of-the-art architecture [12, 16, 24]. Though this was achieved with only 0.8 million trainable parameters and so is a light model that can be used in computers with small resources for traffic sign recognition. This high accuracy can be justified by the pre-processing techniques that were used before training and the robustness of the CNN.

This paper was further extended to recognize traffic signs in real time. The dataset used is the GTSRB dataset which was originally designed for the recognition of 3 super classes, i.e., prohibitory, mandatory, and danger signs. The dataset consists of 600 images for training and 300 images for testing. There is an average of 200 images per category in training. Our goal was not only to detect the 3 super classes but all 43 classes of the German traffic sign using the same dataset. As such, we had an average of 14 images per class for training. Faster R–CNN weights that were used to train on the COCO dataset were then fine-tuned into training for the recognition of traffic signs. The fine-tuned parameters and process is explained in section 3 E. Fine-tuning enabled the model to take less time to train. And the Faster R–CNN achieved a mean average precision (mAP) of 43.26% at a frame rate of 6 fps. Another model that was also investigated for the recognition task is the YOLOv4 [18] model. This model was fine-tuned for traffic sign detection. YOLOv4 achieved a mean average precision (mAP) of 59.88% at a frame rate of 35 fps.

The comparisons between these models were done and some results are shown in Figure 9. The YOLOv4 model was able to detect and classify the different German traffic signs despite using a very small amount of data for training. Other data augmentation techniques could be used to increase the number of training datasets and lead to a better performance of the models.

## Declarations

### Author contribution statement

Njayou Youssouf: Conceived and designed the experiments; Performed the experiments; Analyzed and interpreted the data; Contributed reagents, materials, analysis tools or data; Wrote the paper.

### Funding statement

This research did not receive any specific grant from funding agencies in the public, commercial, or not-for-profit sectors.

### Data availability statement

Data associated with this study has been deposited at https://drive.google.com/drive/folders/1DHnRnM4B0qCHOZaexG30kAScKHT7HD2o?usp=sharing

### Declaration of interest’s statement

The authors declare no conflict of interest.

### Additional information

No additional information is available for this paper.
